# Surgical Management of Thoracic Osteomyelitis due to Escherichia Coli Sepsis

**DOI:** 10.1155/2020/8847504

**Published:** 2020-08-27

**Authors:** Mark K. Lyons, Maziyar Kalani, Matthew T. Neal, Naresh P. Patel

**Affiliations:** Department of Neurological Surgery, Mayo Clinic Arizona, 5777 East Mayo Boulevard, Phoenix, AZ 85054, USA

## Abstract

*Case Report*. *Escherichia coli* is a rare cause of vertebral osteomyelitis. It is more common in adults and males. We present a case of an immunocompetent adult male presenting with a several month history of progressive systemic symptoms and subsequent neurologic compromise. We discuss the neurosurgical evaluation of a patient with a progressive vertebral osteomyelitis and treatment options. Surgical debridement and spinal stabilization were performed and confirmed the diagnosis. The patient successfully completed a prolonged antimicrobial therapy course. The patient made a complete neurologic recovery. We discuss the presentation of a patient with *Escherichia coli* vertebral osteomyelitis and the successful surgical management.

## 1. Introduction

Spinal osteomyelitis, while a relatively uncommon occurrence, has seen an increase in prevalence in part due to increasing spinal surgeries, immunosuppression, drug abuse, and an aging population [1]. *Staphylococcus aureus* (*S aureus*) and *Escherichia coli* (*E coli*) are the most common bacterial pathogens [2–4]. Spontaneous vertebral osteomyelitis is generally considered to be due to hematogenous spread in most cases. Postoperative infections are usually the result of direct inoculation [3, 5]. Cases of *E coli* osteomyelitis are more common in older patients and often associated with urinary tract infections [4–8]. We present the case of an immunocompetent male who developed progressive vertebral osteomyelitis due to *E coli* after a prostate biopsy requiring surgical debridement and spinal stabilization as the definitive treatment.

## 2. Case Report

**Figure 1:**
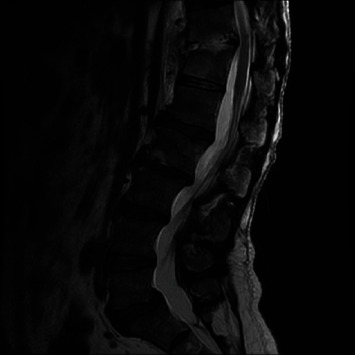
MR thoracic spine T2-weighted image demonstrates mild enhancement in the T11-T12 disc space with mild endplate erosion. No significant spinal stenosis.

**Figure 2:**
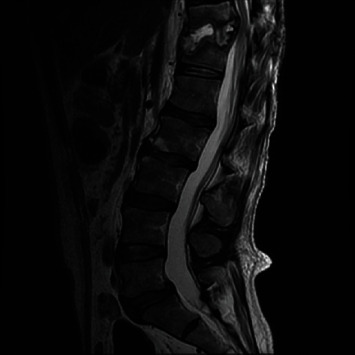
MR thoracic spine T2-weighted image 3 months later demonstrates progression of end plate destruction and phlegmon and progression of spinal stenosis.

**Figure 3:**
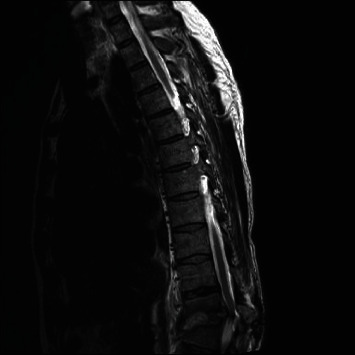
MR thoracic spine T2-weighted image 1 month later showing progressive collapse of T11 and T12 vertebral bodies and severe spinal stenosis.

A 64-year-old man underwent a prostate biopsy and subsequently developed fevers and chills 24 hours later. Urinary and blood cultures were positive for *E coli*, and he was treated with intravenous antibiotics for four days with resolution of his systemic symptoms. Three months later, he presented back to his outside institution with progressive low back pain without neurologic deficits. A magnetic resonance (MR) imaging study was worrisome for T11-T12 osteomyelitis (Figures 1–2). His laboratory studies revealed slightly elevated white blood cell count (WBC) of 12.7 (range 3.4-9.6 mg/L), erythrocyte sedimentation rate (ESR) of 27 (range 0-20 mm/hr), and C-reactive protein (CRP) of 11.4 (range 0-3 mg/L). The patient was started empirically on cefepime for 18 days. The patient reported improvement in his back pain of approximately 50% following this treatment. He remained neurologically intact. A follow-up MR three months later demonstrated progression of osteomyelitis with increased collapse of the vertebral bodies. He was recommended to undergo a computerized tomography (CT)-guided disc space biopsy that was not completed. One month later, he presented to our emergency department at our institution for the first time with severely progressive back pain, high fever, and sepsis. His inflammatory markers had significantly increased. His neurologic exam was limited by severe pain. Blood cultures were positive for *E coli.* Chest X-ray demonstrated pleural effusion. Given his sepsis, the patient was started on vancomycin and piperacillin-tazobactam. MR demonstrated progression of T11 and T12 disc destruction with collapse and severe spinal canal compromise (Figure 3).

**Figure 4:**
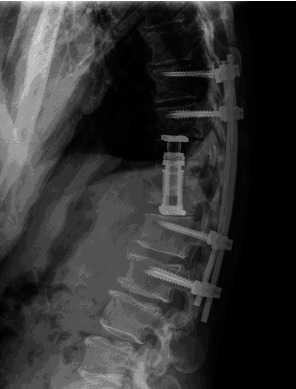
Lateral thoracic spine shows T9-L2 posterior pedicle screw and rod fixation with T11 and T12 corpectomies with expandable intervertebral cage.

**Figure 5:**
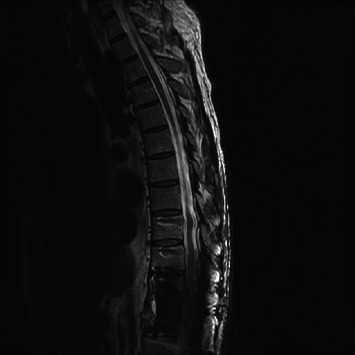
MR thoracic spine T2-weighted image 2 months postoperatively shows decompression of the spinal cord with instrumentation.

The patient was taken to the operating room and underwent a right lateral approach for T11 and T12 corpectomies with implantation of an expandable cage and bilateral T9-L2 percutaneous pedicle screw fixation (Figure 4). Intraoperative cultures confirmed *E coli* osteomyelitis. The organism demonstrated multiple drug resistance. The patient was treated with a six-week course of intravenous ertapenem after which he was started on ongoing suppression with trimethoprim-sulamethoxazole. Clinically, his back pain was resolved with no residual neurologic sequelae. Inflammatory markers returned to normal. Follow-up MR imaging six months after surgery demonstrated resolution of canal compromise and stabilization of thoracolumbar spine (Figure 5).

## 3. Discussion

Vertebral osteomyelitis comprises approximately 4% of the case of osteomyelitis [1, 2, 4, 5]. Adults are much more often afflicted with vertebral osteomyelitis than children. Males more affected and medical comorbidities including immunosuppression, diabetes, and neoplastic disease predispose patients to vertebral osteomyelitis [2, 3, 7]. The presenting symptoms are generally nonspecific but commonly include progressive back pain and fever. Laboratory studies including CRP, ESR, and elevated WBC are common findings. Spinal radiographs may not demonstrate significant abnormalities early on in the process. Magnetic resonance imaging with gadolinium is the imaging study of choice to identify the infectious process. The sensitivity is reported to be 96%, and the specificity is 93% in discerning particularly between infectious and neoplastic processes [3].

Blood cultures as part of the work-up should be included but can often be negative. In patients without neurologic deficits, significant neural compression and no impending structural instability can undergo biopsy of the area of abnormality for diagnosis. If initially negative, these studies should be repeated. In our case, the patient's symptoms had progressed with significant structural compromise. He was septic, and the decision was made to proceed with definite surgery to evacuate the source of infection and stabilize the spinal column in order to preserve neurologic function and optimize antimicrobial therapy.

The most common bacterial pathogen resulting in spinal osteomyelitis is *Staphylococcus aureus*. Vertebral osteomyelitis due to *E coli* is rare [3, 4, 6, 7]. The most common source for *E coli* osteomyelitis is secondary to genitourinary conditions [7]. Our patient had undergone a prostate biopsy 24 hours prior to developing fevers and chills. Unfortunately, several months passed with progressing symptoms until he was referred for definitive evaluation and treatment. Progressive systemic symptoms of fever, pain, and radiographic destructive progression should prompt urgent intervention. Our case demonstrated an aggressive pathogen that was resistant to multiple antibiotics. Patients with vertebral osteomyelitis can have a successful outcome when appropriate treatment is instituted.

## 4. Conclusions

*Escherichia coli* is a rare cause of vertebral osteomyelitis. Our patient was not immunocompromised and did not have other risk factors associated with the development of osteomyelitis. However, the history of a urologic procedure immediately prior to the development of systemic symptoms and ultimately neurologic symptoms should alert the clinician to the possibility of vertebral osteomyelitis. Aggressive surgical debridement, spinal stabilization, and appropriate antimicrobial therapy can result in successful patient outcomes.
